# Management of osteochondritis dissecans lesions of the elbow including return to sport remains variable among orthopaedic surgeons

**DOI:** 10.1007/s00402-024-05635-5

**Published:** 2024-12-16

**Authors:** Eric N. Bowman, Gabriel Lane, Charles F. Goldfarb, Matthew V. Smith

**Affiliations:** 1 Department of Orthopaedics, Vanderbilt University Medical Center, 1215 21st Avenue South, 4200 Medical Center East, Nashville, TN 37232-8774 USA; 2 Meharry Medical College, 1005 Dr. DB Todd Jr. Blvd, Nashville, TN 37208 USA; 3 Orthopedic Surgery, Division of Hand and Microsurgery, Washington University in St. Louis, 14532 South Outer Forty Drive, Chesterfield, MO 63017 USA; 4 Washington University in St. Louis, 14532 South Outer Forty Drive, Chesterfield, MO 63017 USA

**Keywords:** Capitellum, OCD, Osteochondral, Osteochondritis dissecans, Baseball, Elbow

## Abstract

**Introduction:**

Management of osteochondritis dissecans (OCD) lesions of the capitellum is challenging. Historically, variability exists between surgeons in the evaluation, treatment, and return to sport criteria. The purpose of this study was to define the current trends regarding evaluation, nonoperative and surgical management, and return to sport criteria for capitellar OCD lesions among surgeons.

**Methods:**

A 21-question cross-sectional survey was administered to 24 Orthopaedic surgeons specializing in elbow OCDs. The survey included questions concerning imaging, specific non-operative treatments trialed, indications for surgery for stable and unstable lesions, preferred surgical techniques, osteochondral autograft utilization, and factors determining return to sport.

**Results:**

Twenty-one surgeons responded (88%). The most common surgical indications for stable lesions were time (≥ 6 months, 68%) and mechanical symptoms (52%). Drilling (45%) and fragment fixation (35%) were most preferred. For unstable lesions, factors in order of importance for determining surgical procedure were lesion size, lateral wall integrity, location on capitellum, skeletal maturity, and sport. For small (< 1 cm^2^), centralized lesions, 81% preferred debridement with microfracture. For large (> 1 cm^2^), lateralized lesions, 52% preferred debridement and microfracture and 48% preferred osteochondral autograft transfer (OAT). OAT was considered for 80% of failed procedures, 47% with lateral wall involvement, and 27% > 1 cm^2^. Return to sport after debridement was typically 2–3 months (52%), fragment fixation was 4 months (52%), and OAT was 4–6 months, while microfracture had wide variability (3–6 months). The factors in order of importance were lack of pain, time, then imaging. Two-thirds of surgeons wait longer to release overhead athletes or gymnasts.

**Conclusions:**

There is significant variability in the management of capitellar OCD in athletes. Small, centralized lesions are likely to be treated with debridement and microfracture with faster return to sport. Treatment of large, lateral lesions remains variable. Regarding OAT procedures, perceived morbidity, reimbursement, and limited evidence dissuade use. There is no consensus on return to sport, though lack of pain and time were most important; overhead athletes and gymnasts are restricted longer from returning to sport.

**Level of Evidence**

Level 5, diagnostic, cross-sectional survey.

## Introduction

Osteochondritis Dissecans (OCD) of the capitellum commonly affects adolescent baseball players and gymnasts, with a cumulative incidence of 1.8% per year [10, 15]. The pathology was described by König in 1888 when treating adolescent elbows for loose bodies [12]. OCD lesions are acquired deficiencies of the subchondral bone with or without involvement of overlying articular cartilage [5, 7]. The pathophysiology continues to be elucidated, however, repetitive elbow stress under valgus load is commonly implicated, in addition to biologic factors (likely vascularity) near the epiphysis [4, 5, 7].

It frequently occurs in adolescents and may be an incidental imaging finding or present with pain or mechanical symptoms impairing athletic performance [5]. One-third of elbow OCD lesions present without pain [15]. Treatment options vary depending on patient age and lesion characteristics. The goal of treatment is to restore full, painless range of motion in order to return the athlete to their previous level of performance. Patients with open capitellar growth plates have higher success rates with conservative treatment including rest and physical therapy [23].

Surgical options for OCD lesions include removal of loose bodies, debridement, drilling, microfracture, chondrocyte implantation, and osteochondral auto- or allograft transplantation (OAT). Indications for one treatment over another have not been clearly defined. As such, there is high variability in the treatment patterns of surgeons. The purpose of this study was to define the current trends regarding evaluation, nonoperative and surgical management, and return to sport criteria for capitellar OCD lesions among surgeons.

## Materials and methods

A cross-sectional survey was performed to evaluate surgeon treatment trends in March 2021. Twenty-four surgeons were identified based on their treatment of patients with elbow capitellar OCD lesions, primarily through colleague referral and publications regarding capitellar OCD lesions. Participants were approached based on their involvement with elbow OCD research groups within the American Academy of Orthopaedic Surgery. A 21-question anonymous Research Electronic Data Capture (REDCap) (Nashville, TN) survey was administered after consent was provided. This study was approved and monitored by the Institutional Review Board #210080.

**Fig. 1:**
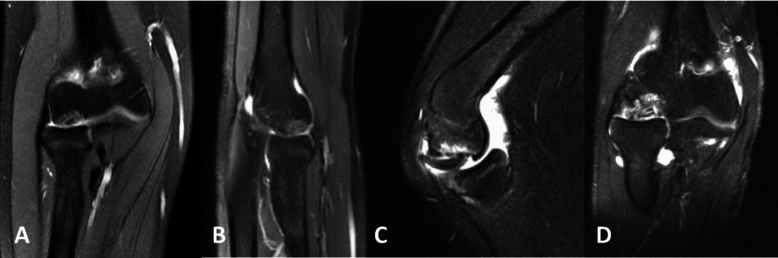
MRI examples of unstable osteochondritis dissecans lesions of the capitellum. **A** Coronal image of a small (< 1 cm^2^), centralized lesion. **B** Sagittal image of a small (< 1 cm^2^), centralized lesion. **C** Sagittal image of a large (> 1 cm^2^) lesion. **D** Coronal image of a large (> 1 cm^2^) lesion

Demographic information was collected from each surgeon including the number of capitellar OCD lesions treated per year and primary specialty. Regarding OCD elbow lesions, queries were made concerning typical imaging utilized, specific non-operative treatments trialed, indications for surgery for stable and unstable lesions, preferred surgical techniques, osteochondral autograft use, and factors utilized in determining return to sport. We defined lesions according to the Dipaola [6] and International Cartilage Repair Society (ICRS) guidelines as: stable lesion: normal elbow ROM, flattening or lucency of the subchondral bone on radiograph without fragmentation, immature capitellum with open physis OR Unstable lesion: (at least one of the following): loss of elbow motion > 20 degrees, fragmentation, or mature capitellum with closed physis [2, 23]. Regarding unstable OCD, we further classified lesions as small (< 1 cm^2^) and central or large (> 1 cm^2^) (Fig. 1). A description of available treatment options available for OCD lesions of the capitellum is available in Table 1. Survey questions are in Table 2 and the complete survey is located in Addendum 1. All analyses including descriptive statistics were conducted in Excel 2019 (Microsoft, Redmond, WA).

**Table 1:** A description of available treatment options available for OCD lesions of the capitellum

Treatment option	Description	Indications
Observation	Monitor physical exam and imaging	Stable lesion
Debridement	Removal of loose or unstable fragments	Unstable lesion, chondral flap
Subchondral drilling	Marrow stimulation via ante-or retrograde drilling	Stable lesion
Fragment fixation	Various osteochondral fixation devices	Unstable lesion with viable cartilage and bone fragment
Microfracture	Marrow stimulation in base of chondral defect	Unstable lesion with chondral defect
Osteochondral grafting (OATS)	Osteochondral graft transfer from donor site (auto- or allograft)	Large unstable lesion, lateral wall involvement, revision procedure

**Table 2:** Survey questions

1. On average, how many patients do you treat per year with capitellar OCD lesions?
2. What is your primary orthopaedic subspecialty?
3. How often do you obtain the advanced imaging modalities for the evaluation of capitellar OCD lesions (CT, MRI, MRA, ultrasound)?
4. What surgical approach do you most often utilize in the treatment of an OCD of the capitellum? 5. How often do you recommend non-operative treatments for stable OCD lesions?
6. On what basis in the care of a stable lesion do you consider surgical intervention?
7. If you consider surgical intervention of a stable lesion based on length of symptoms, how long do you wait?
8. What is your preferred treatment for a stable lesion which has failed non-operative treatment?
9. In an unstable appearing lesion, how long do you recommend a trial of nonoperative treatment?
10. What is your most common treatment for small (< 1 cm^2^), central, unstable lesions (assuming the fragment cannot be fixed)?
11. What is your most common treatment for large (> 1 cm^2^), unstable lesions (assuming the lesion cannot be fixed)?
12. Please rank the following factors and importance for guiding treatment of unstable capitellar OCD: Lesion size, location, lateral wall integrity, skeletal maturity, type of sport
13. Do you utilize osteochondral graft transfers (auto or allograft) in the treatment of OCD of the capitellum?
14. If you utilize osteochondral graft transfers, when did you consider them?
15. If you do not use osteochondral grafts, why not?
16. In patients with stable OCD lesions, how important are the following factors in determining return to sports (time, pain, advanced imaging)?
17. If based on time, how long delay before allowing return to sport? 18. If based on advanced imaging, which modality to use before allowing return to sport? 19. After surgical treatment of a capitellar OCD lesion, do routinely repeat advanced imaging before allowing patients to return to sport? 20. For each treatment type, when to allow return to sport? 21. Do adjust to return to sport timeframe based on type of sport?

For complete survey please refer to Addendum 1

*OCD* osteochondritis dissecans, *CT* computed tomography, *MRI* magnetic resonance imaging, *MRA* magnetic resonance arthrogram

## Results

We emailed surveys to 24 orthopaedic surgeons and 21 responded completely (88%). Most respondents had advanced fellowship training and certification in sports medicine (66.7%). The mean number of OCD cases per year was 12 and the median was 10.

### Imaging and nonoperative treatment

Magnetic resonance imaging (MRI) was the most selected modality, with 86% of surgeons always and 14% sometimes obtaining. Magnetic resonance arthrogram (MRA) was obtained sometimes by 67% and always by 10% of surgeons. Computed tomography (CT) was sometimes used by 62% and never used by 33% of surgeons.

Non-operative treatment for stable lesions most commonly included activity modification or restriction from sports (86%), followed by range of motion (71%), and formal physical therapy (PT) (38% always and 62% sometimes). Elbow bracing was always used by 10% and never used by 42% of surgeons. Orthobiologics were sometimes used by 29%. Surgeon demographics, imaging, and treatment details are found in Table 3.

**Table 3:** Surgeon demographics, preferred surgical approach, imaging preferences, and treatment preferences for stable OCD lesions among 21 survey respondents

	*n*	%	Always *n* (%)	Sometimes *n* (%)	Never *n* (%)
Surgeon demographics					
OCDs treated per year					
< 10	11	52			
10–15	5	24			
15–20	3	14			
20+	2	10			
Primary specialty					
Sports medicine	14	67			
Shoulder/elbow	4	19			
Hand	3	14			
Surgical approach					
Arthroscopic	18	86			
Open	3	14			
Imaging preferences					
CT scan			1 (5)	13 (62)	7 (33)
MRI			18 (86)	3 (14)	0 (0)
MRA			2 (10)	14 (67)	5 (24)
Ultrasound			0 (0)	0 (0)	21 (100)
Treatment					
Non-operative					
Rest			18 (86)	3 (14)	0 (0)
Stretching/motion			15 (71)	6 (29)	0 (0)
Formal PT			8 (38)	13 (62)	0 (0)
Elbow brace			2 (10)	10 (48)	9 (42)
Orthobiologics			0 (0)	6 (29)	15 (71)
Operative indication—*stable* lesion					
Mechanical symptoms			11 (52)	10 (47)	0 (0)
Loss of motion			5 (24)	16 (76)	0 (0)
Time based (*n* = 19)			7 (33)	12 (57)	2 (10)
3 months	5	26			
6 months	14	74			

*OCD* osteochondritis dissecans, *CT* computed tomography, *MRI* magnetic resonance imaging, *MRA* magnetic resonance arthrogram, *rest* activity modification/restriction from sports, *orthobiologics* platelet rich plasma or bone marrow aspirate concentrate injections, *time based* no evidence of healing clinically or on imaging

### Surgical management

Surgical indications for stable lesions were variable, but mechanical symptoms were always an indication for 52% of the respondents, while loss of motion was always an indication for 24%. Duration of symptoms (time) was always a factor in determining the need for surgery for 33% of the respondents, while only 10% indicated that time was never a factor. Six months without evidence of clinical or radiographic healing was the most common time-based indication for surgery (68%).

**Fig. 2:**
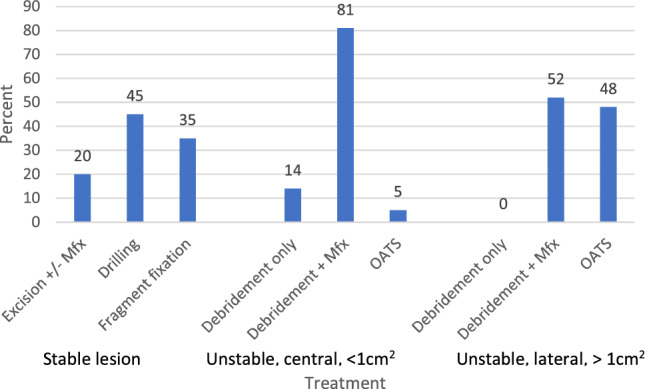
Distribution of surgical treatment of capitellar OCD lesions. *Excision* fragment excision with or without marrow stimulation or microfracture (Mfx), *Drilling* antegrade or retrograde, *OATS* osteochondral graft transfers (autograft or allograft)

For unstable lesions, 62% of the respondents proceed to surgery once the defect is identified as unstable. The remaining participants based their decision on other factors (OCD lesion size, location, etc.). Time from onset of presentation did not play a role for any surgeon in an unstable lesion. Factors in order of importance for determining treatment of unstable OCD lesions were lesion size, integrity of the lateral wall, location on the capitellum (lateral or central), skeletal maturity, and type of sport (gymnast vs. overhead athlete). For surgical approach, 86% preferred an arthroscopic approach unless grafting was performed. The surgical management breakdown of by type of lesion is in Fig. 2.

### Osteochondral grafting

For 80% of the respondents, osteochondral grafting (auto or allograft—OAT) procedures were “always considered” for failed debridement or marrow stimulation procedures. Osteochondral graft was “always considered” for lesions involving the lateral wall of the capitellum by 47% of the respondents. Osteochondral graft was “always considered” for lesions larger than 1 cm^2^ by 27% of the respondents. Otherwise, OAT procedures were “sometimes considered” for these indications by the remainder of participants. “Osteochondral autograft only” usage was reported by 38%, “allograft only” by 5%, and either “auto or allograft” by 29%; 29% of surgeons did not report using OATs. The cited reasons for not using OATs were limited evidence in the literature and associated morbidity (both 67%), reimbursement concerns (60%), and lack of familiarity with the procedure (50%).

### Return to sport

The most important factor in determining return to sport after non-operative treatment for a stable lesion was the patient being pain free, as 81% deemed this very important. Time was somewhat important for 57% and very important for 33%. The most common time duration before allowing return to sport was 3–4 months (53%), followed by 5–6 months (21%), and 1.5–3 months (16%). When the surgeon obtained advanced imaging to assist in determining return to sport, MRI was preferred; the MRI demonstrating a healed lesion was considered very important (48%) or somewhat important (43%) in decision-making. However, after surgical treatment, only 14% obtain an MRI to assess healing, typically at 3–6 months.

**Fig. 3:**
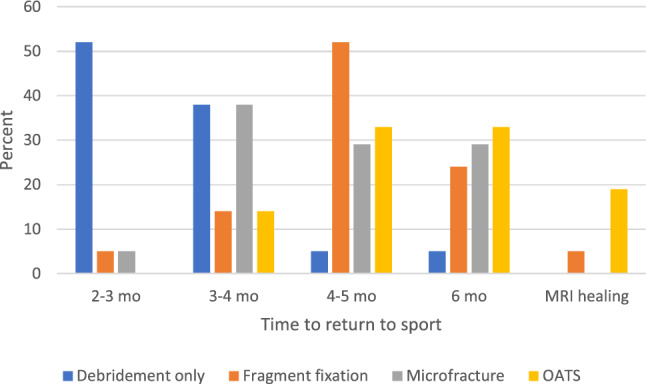
Return to sport based on procedure performed

Return to sport after debridement is most commonly 2–3 months (52%). For fragment fixation, the median is 4–5 months (52%). After microfracture, there is a wide distribution: 3–4 months (38%), 4–5 months (29%), and 6 months (29%). Return to sport after OAT was typically 4–6 months with repeat MRI obtained by 19%. Two-thirds of surgeons wait longer for overhead athletes or gymnasts. Complete return to sport data found in Fig. 3.

## Discussion

This study highlights the variability that currently exists in the evaluation, management, and return to sport following capitellar OCD management. Small, central, unstable lesions have the highest agreement regarding treatment, with 81% preferring debridement with microfracture. The treatment of large, lateral, unstable lesions were split between debridement with microfracture and OAT. Those less likely to perform OAT cite lack of evidence in the literature, reimbursement concerns, and associated morbidity as factors. There is no consensus on return to sport, though lack of pain and time were most important.

### Imaging

Radiographic evaluation is the initial imaging modality in the work-up of a painful elbow, with findings ranging from “normal” to lucency below the articular surface to loose body formation [2, 23]. CT may be useful in further identifying lesion location, stability, loose bodies, and bony integration post-operatively [21]. Though heavily operator dependent, ultrasound has been shown to be beneficial in determining lesion stability [24]. MRI is the most utilized diagnostic modality in our cohort, 86% always obtain, with 67% occasionally obtaining MRAs. Utilizing appropriate criteria, sensitivity and specificity for stability on MRI may approach 100% [11]. An area for further research is defining the imaging characteristics that may provide support for one treatment over another, such as lesion location and integrity of the lateral wall.

### Nonoperative management

In this study, for stable lesions, activity modification was highly recommended, with variable integration of elbow and shoulder mobility, formal physical therapy for range of motion, and bracing or immobilization. Orthobiologics (i.e., platelet-rich plasma) were utilized by 29%. While there is not strong evidence for a specific protocol in the literature, rest/activity modification until resolution of symptoms appears to be essential, which may take 3–6 months [9, 23]. For stable lesions, non-operative treatment may be effective in 80–90% of compliant patients, with worse outcomes reported in those who continue aggressive activity [9, 16, 23]. The role of bracing or immobilization is less clear, and those who favor it cite evidence from the knee OCD literature which demonstrated greatly improved healing with casting and non-weightbearing [26]. Patients with open physes and smaller lesions without cyst-like changes or radial head enlargement have the best prognosis [8, 18].

### Operative management

For stable lesions not improving within 6 months via conservative measures and for unstable lesions, surgery is often indicated [9]. If the articular surface is stable, subchondral drilling is a reasonable option to incur marrow stimulation and subsequent healing. Fragment fixation is ideal if the lesion is amenable, with excellent outcomes [25]. The most important factors for determining treatment of unstable OCD lesions according to our surgeons were lesion size, lateral wall integrity, and location (central or lateral) on the capitellum. There are no comparative studies to evaluate treatment methods for unstable lesions, but short-term outcomes are generally good to excellent regardless of surgical treatment [13, 20]. Debridement of unstable lesions alone demonstrated a 10-year survivorship of 88% [1, 17]. Central lesions portend a better prognosis; large lesions (> 1 cm^2^) and those involving the lateral wall have a poorer prognosis [3, 14, 22].

OAT procedures have emerged as a reliable treatment option for large, unstable lesions or for salvage procedures. They demonstrate good outcomes with high-satisfaction and return to sport, few complications, and low-donor site morbidity [14, 19]. A systematic review by Sayani et al., however, demonstrated equivalent outcomes of OATs vs fragment fixation for high-grade lesions regardless of lesion location [20]. Currently, there remains a paucity of long-term data to guide treatment. For this reason, reimbursement for OAT procedures remains variable, as some insurers still define it as “experimental and investigational.” Procedures vary greatly in terms of cost and time to return to sport. As demonstrated by the variability in the literature as well as our surgeon cohort, further studies are required to better define appropriate treatment options based on lesion characteristics.

### Return to sport

There were no consensus return to sport criteria among the surgeons surveyed. Lack of pain and restored range of motion were the most agreed upon. Time was the second most important factor but was variable depending on the surgeon and procedure. Repeat MRI post-operatively was not commonly utilized, however. Treatment of stable lesions with conservative treatment yields nearly 90% return to sport [20]. Unstable lesions treated surgically have > 80% return to sport, however, only two-thirds of athletes return to their previous level [20]. In one study, following loose body removal and drilling/microfracture, 86% of patients returned to sport (67% to primary sport) [13]. Fragment fixation demonstrated a modest return to sport of 60–100%, but only 69% returned to their previous level of play in one study [25]. Finally, Sasanuma et al. showed that 94% of patients treated with OAT returned to baseball, but only 56% continued pitching and demonstrated a lower satisfaction due to pain while pitching [19]. This is likely due to increased lateral compressive stress during pitching. One systematic review found an overall surgical return to sport rate of 86% at a mean of 5.6 months, with OATs (94%) higher than debridement (71%) and fixation (64%) [27]. Future studies should focus on objective criteria that can guide evidence-based return to sport.

### Limitations

One limitation of this study is that surgeons were selected through referral or academic recognition, therefore, results may be biased. Even so, the rarity of the condition is highlighted in the fact that over half of these “experts” perform less than 10 procedures per year. Also, treatment decisions are determined after a complex, personalized conversation with each patient and thus other factors not evaluated in this survey may impact treatment. Although low numbers prevent correlation of treatment with specific surgeon characteristics including volume, practice setting, and training, this would be an interesting area for future analysis. Clinical vignettes with patient imaging may be a better way to understand the interactions of these factors.

## Conclusions

There is significant variability in the management of capitellar OCD in athletes. Small, centralized lesions are likely to be treated with debridement and microfracture with faster return to sport. Treatment of large, lateral lesions remains variable. Regarding OAT procedures, perceived morbidity, reimbursement, and limited evidence dissuade use. There is no consensus on return to sport, though lack of pain and time were most important; overhead athletes and gymnasts are restricted longer from returning to sport.
